# Spatial Analysis of Neural Cell Proteomic Profiles Following Ischemic Stroke in Mice Using High-Plex Digital Spatial Profiling

**DOI:** 10.1007/s12035-022-03031-x

**Published:** 2022-09-24

**Authors:** Jessica M. Noll, Catherine J. Augello, Esra Kürüm, Liuliu Pan, Anna Pavenko, Andy Nam, Byron D. Ford

**Affiliations:** 1 Division of Biomedical Sciences, University of California-Riverside School of Medicine, 900 University Ave, Riverside, CA 92521 USA; 2 Division of Bioengineering, University of California, 900 University Ave, Riverside, CA 92521 USA; 3 Department of Statistics, University of California, 900 University Ave, Riverside, CA 92521 USA; 4 Nanostring Technologies, Seattle, WA 98109 USA

**Keywords:** Autophagy, Digital spatial profile, Inflammation, Ischemia, Proteomics, Stroke

## Abstract

Stroke is ranked as the fifth leading cause of death and the leading cause of adult disability in the USA. The progression of neuronal damage after stroke is recognized to be a complex integration of glia, neurons, and the surrounding extracellular matrix, therefore potential treatments must target the detrimental effects created by these interactions. In this study, we examined the spatial cellular and neuroinflammatory mechanisms occurring early after ischemic stroke utilizing Nanostring Digital Spatial Profiling (DSP) technology. Male C57bl/6 mice were subjected to photothrombotic middle cerebral artery occlusion (MCAO) and sacrificed at 3 days post-ischemia. Spatial distinction of the ipsilateral hemisphere was studied according to the regions of interest: the ischemic core, peri-infarct tissues, and peri-infarct normal tissue (PiNT) in comparison to the contralateral hemisphere. We demonstrated that the ipsilateral hemisphere initiates distinct spatial regulatory proteomic profiles with DSP technology that can be identified consistently with the immunohistochemical markers, FJB, GFAP, and Iba-1. The core border profile demonstrated an induction of neuronal death, apoptosis, autophagy, immunoreactivity, and early degenerative proteins. Most notably, the core border resulted in a decrease of the neuronal proteins Map2 and NeuN; an increase in the autophagy proteins BAG3 and CTSD; an increase in the microglial and peripheral immune invasion proteins Iba1, CD45, CD11b, and CD39; and an increase in the neurodegenerative proteins BACE1, APP, amyloid β 1–42, ApoE, and hyperphosphorylated tau protein S-199. The peri-infarct region demonstrated increased astrocytic, immunoreactivity, apoptotic, and neurodegenerative proteomic profiles, with an increase in BAG3, GFAP, and hyperphosphorylated tau protein S-199. The PiNT region displayed minimal changes compared to the contralateral cortex with only an increase in GFAP. In this study, we showed that mechanisms known to be associated with stroke, such as apoptosis and inflammation, occur in distinct spatial domains of the injured brain following ischemia. We also demonstrated the dysregulation of specific autophagic pathways that may lead to neurodegeneration in peri-infarct brain tissues. Taken together, these data suggest that identifying post-ischemic mechanisms occurring in a spatiotemporal manner may lead to more precise targets for successful therapeutic interventions to treat stroke.

## Introduction

Ischemic stroke is the leading cause of serious long-term disability and the 5th leading cause of death in the USA [1]. Ischemic stroke represents approximately 80% of strokes and presents as the occlusion or blockage of an artery within the brain [1]. Currently, only tissue plasminogen activator (tPA) is approved for treatment of stroke, but has a limited time window for therapeutic use and is only administered to 3–5% of patients [2, 3]. However, tPA treatment does not play a neuroprotective role, and is not ideal for prevention of secondary, long-term neurodegenerative damage. Additionally, as stroke progression is recognized to be a complex integration of glia, neurons, and the surrounding extracellular matrix, treatments should aim to directly target the detrimental effects created by these interactions [4–6]. Therefore, there is a strong need to understand the cellular and molecular mechanisms occurring early after ischemic stroke in a spatial manner in order to develop effective ischemic stroke treatments.

Ischemic stroke begins with loss of glucose and oxygen that triggers necrotic neuronal death (infarct), release of oxygen radicals, microvascular injury, and blood–brain barrier disruption in the ischemic core [5]. This then leads to secondary apoptotic, excitatory neuronal death in the surrounding ischemic penumbra with increases in neuroinflammation that can be prolonged for days after injury [7, 8]. After ischemic damage is complete, the remaining, bordering region of reversibly damaged cells in an area of decreased neuronal density is defined as the peri-infarct tissue [9]. The peri-infarct region exhibits a complex, dynamic interaction between immune and neuronal cells in both rodents and humans that critically determines late neuroprotection and repair processes after ischemia [10, 11]. Neuroinflammation after ischemia involves activation of microglia, astrocytes, and infiltration of peripheral leukocytes [12–15]. Resident microglia demonstrate reactivity with a change in morphology and infiltration into the core [15–17]. Astrocytes proliferate and increase expression of inflammatory factors such as glial fibrillary acidic protein (GFAP) which can later lead to astroglial scar formation along the infarct border [14, 16, 17]. These interactions become even more complex with the understanding that peri-infarct regions in relationship to the core lesion demonstrate distinct mechanisms in a spatial manner. The core region demonstrates progressive cavitation, while the peri-infarct tissue becomes increasingly plastic. Thus, understanding the complexities of these interacting systems as they develop after ischemia in a spatial manner is key to further elucidating the underlying mechanisms for future preventions and treatments.

Gene and protein profile expressions have often been analyzed after ischemic stroke to elucidate the molecular mechanisms during various timepoints [18–26], but have been limited by technological challenges. Technology limitations have included the requirement that tissue be analyzed in whole sections, such as the entire ipsilateral and contralateral hemispheres. Some studies have carefully extracted cortices and subcortices from each hemisphere with more specific profile success. However, the spatial profile specific to the early repair mechanisms occurring in these distinct regions post-ischemia: the ischemic core, peri-infarct, and peri-infarct normal tissue (PiNT) have not yet been clearly elucidated in much detail. In this study, we examined the spatial regulation of protein profiles initiated at 3 days post-ischemia in order to visualize the critical time point where endogenous early neuroprotection effects taper off and neural repair begins to initiate [4]. By utilizing immunohistochemistry and Nanostring GeoMx Digital Spatial Profiling (DSP), we analyzed panels of proteins in regions of interest including the ischemic core border, peri-infarct, and PiNT at 3 days post-ischemia compared to regions in the contralateral cortex. NanoString’s GeoMx DSP technology generates profiling data for validated protein analytes using high-plex spatial profiling to rapidly and quantitatively assess the biological implications of the heterogeneity within the tissue samples [27, 28]

We demonstrated that the ipsilateral hemisphere particularly initiated distinct proteomic regulatory profiles in a spatial manner that can be co-localized consistently with the cellular markers, fluoro Jade B (FJB), GFAP, and Iba-1. Additionally, the core border region presented a unique proteomic profile that represents cellular death, immunoreactivity, and early degeneration. Most notably, the core border resulted in a decrease of the neuronal proteins Map2 and NeuN; an increase in the autophagic proteins BAG3 and CTSD; an increase in the microglial and peripheral immune invasion proteins Iba1, CD45, CD11b, and CD39; and an increase in the neurodegenerative proteins BACE1, APP, amyloid β 1–42, ApoE, and hyperphosphorylated tau protein S-199. The peri-infarct region demonstrated increased astrocytic immunoreactivity, apoptotic, and neurodegenerative proteomic profile, with an increase in the autophagic protein BAG3, GFAP, and hyperphosphorylated tau protein S-199. The PiNT region displayed minimal changes compared to the contralateral cortex with only an increase in GFAP. These findings may aid in identifying novel therapeutic strategies for the treatment of stroke.

## Materials and Methods

### Animals

All animals used in these studies were treated humanely and with regard for alleviation of suffering and pain, and all protocols involving animals were approved by the IACUC of University of California-Riverside prior to the initiation of experimentation (protocol # AUP 20,190,021). Male and female C57BL6 mice (8–10 weeks old) were purchased from Jackson Laboratories (Cat# #000,664, Bar Harbor, Maine) and housed with a 12-h daily light/dark cycle. Food and water were provided ad libitum. All surgical procedures were performed by sterile/aseptic techniques in accordance with institutional guidelines.

### Photothrombotic Middle Cerebral Occlusion

Animals were randomized and subjected to left photothrombotic MCA occlusion (MCAO) or sham operation as described below. Mice were anesthetized with 2% isoflurane and circulating air (N_2_O:O_2_ at approximately 2:1) and maintained anesthetized during the procedure via a modified gas tubing nose connection on a stereotaxic instrument. Eye lubricant was applied to protect the eyes and body temperature was maintained via a heating pad placed underneath the mice during surgery at 37 °C. MCAO was performed in an adapted accordance to Zhong et al. [29]. Rose Bengal (10 mg/mL; Cat#330,000, Sigma, Burlington, MA) was injected intraperitoneally (i.p.) at 10 mL/g and allowed to incubate for 8 min. The animal was stabilized in the stereotaxic instrument and the scalp hair was removed. The scalp was disinfected with iodine and ethanol, and then followed by a midline skin incision to expose the skull above the left sensorimotor cortex. A 2-mm diameter focal green laser (520 nm; Cat# LP520-MF100 ThorLabs Inc, Newton, NJ) was directed at 2-mm lateral left and 0.6-mm posterior of bregma. Laser irradiation occurred for 20 min at 10 mW. After irradiation, the midline incision was sealed with Vetbond glue (Cat#NC0398332, Fisher Scientific, Hampton, NH) followed by triple antibiotic ointment. Mice were then placed into a 37 °C incubation chamber for 20–30 min to recover. Sham control animals included (1) Rose Bengal injection without laser irradiation and (2) laser irradiation without Rose Bengal injection. Mice were sacrificed at 3 days post-ischemia (dpi). Mice were euthanized by primary method of transcardial perfusion and secondary method of decapitation according to IACUC protocol #20,190,021.

### Histology and Immunohistochemistry

After MCAO, mice were deeply anesthetized with 2% isoflurane and perfused transcardially with saline followed by cold 4% paraformaldehyde (PFA) solution (Cat#HT501128, Sigma, Burlington, MA) (*n* = 4). Brains were quickly removed and maintained in 4% PFA for 24 h. Brains in preparation for histological and immunohistochemical analysis were quickly removed after transcardial perfusion and maintained in 4% PFA for 24 h before being cryoprotected in 30% sucrose/PBS (Sucrose: Cat#C12H22O11, Fisher Scientific, Hampton, NH; PBS: Cat#SH30258.02, HyClone, Logan, UT). The brains were then flash frozen and stored at – 80 °C until sectioning. Coronal sections of 12–15 μm thickness were cryosectioned and mounted on slides which were then stored at – 80 °C until further processed. Adjacent sections were used for correlating stains at each stereotaxic location. Brains from sham animals we used as controls (*n* = 2).

Cresyl violet (Cat#C5042, Millipore, Billerica, MA) stain was first reconstituted from powder with distilled water, allowed to stir overnight, and then 0.3% glacial acetic acid (Cat#A38SI-212, Fisher Scientific, Hampton, NH) was added and mixed thoroughly. Sections were stained with cresyl violet on slides beginning with rehydrating steps of 15 min incubation with 95% ethanol (Cat#EX0276-4, Millipore, Billerica, MA), 1 min with 70% ethanol, 1 min with 50% ethanol, 2 min with distilled water, and 1 min with distilled water. Sections were then stained with cresyl violet warmed to 37.5 °C for 3 min followed by distilled water for 1 min. Sections were then dehydrated with 1 min of 50% ethanol, 2 min of 70% ethanol with 1% glacial acetic acid, 2 min of 95% ethanol, and 1 min of 100% ethanol. After allowing the ethanol to dry off the sections, they were incubated with warmed cresyl violet again for 2 min followed by distilled water for 2 min. Sections were cleared with a 5-min wash of Histoclear (Cat#H2779-1L, Sigma, Burlington, MA) and mounted and cover slipped with DPX (Cat#06,522, Millipore, Billerica, MA).

Fluoro Jade B (FJB; Cat#AG310, Millipore, Billerica, MA) labeling was performed in an adapted protocol according to Noll et al. 2019 to ensure infarct presence and record infarct size and location [26]. Sections were post-fixed with 10% formalin for 10 min and then washed twice with PBS for 5 min. Sections were then directly incubated in 0.06% potassium permanganate (KMnO_4_; Cat#6360–16, Ricca Chemical, Arlington, TX) for 3 min followed by distilled water for 2 min. Sections were then incubated in a freshly prepared solution of 0.0004% FJB with DAPI (Cat#ab228549, Abcam, Waltham, MA) for 20 min, rinsed in distilled water 3 times for 2 min, and then dried at 50 °C.

For immunohistochemical studies, sections were dried at room temperature for 30 min. After rinsing with 0.01 M PBS, sections were blocked in PBS containing 5% normal donkey serum (Cat#ab7475, Abcam, Waltham, MA) and 0.1% Triton X-100 (Cat#X100, Sigma, Burlington, MA) for 1–2 h at room temperature, rinsed with PBS/0.2% Tween-20 (PBST; Cat#01,512, Chem-Impex International, Wood Dale, IL) and then incubated overnight at 4 °C with primary antibodies of polyclonal rabbit anti-Iba-1 (1:1000, Cat#019–19,741, Wako, Osaka, Japan) and Cy3-conjugated monoclonal mouse anti-GFAP (1:400, Cat#C9205, Sigma, Burlington, MA). Sections were washed 3 times with PBST, incubated with respective AlexaFluor594-conjugated donkey anti-rabbit IgG antibody (1:400, Cat#711–585-152, Jackson ImmunoResearch Laboratory, West Grove, PA) for 1 h at room temperature, then rinsed 4 times with PBST before mounting with DAPI-Fluoromount-G (Cat#OB010020, Fisher Scientific, Pittsburgh, PA).

### Immunohistochemical Quantification

**Fig. 1:**
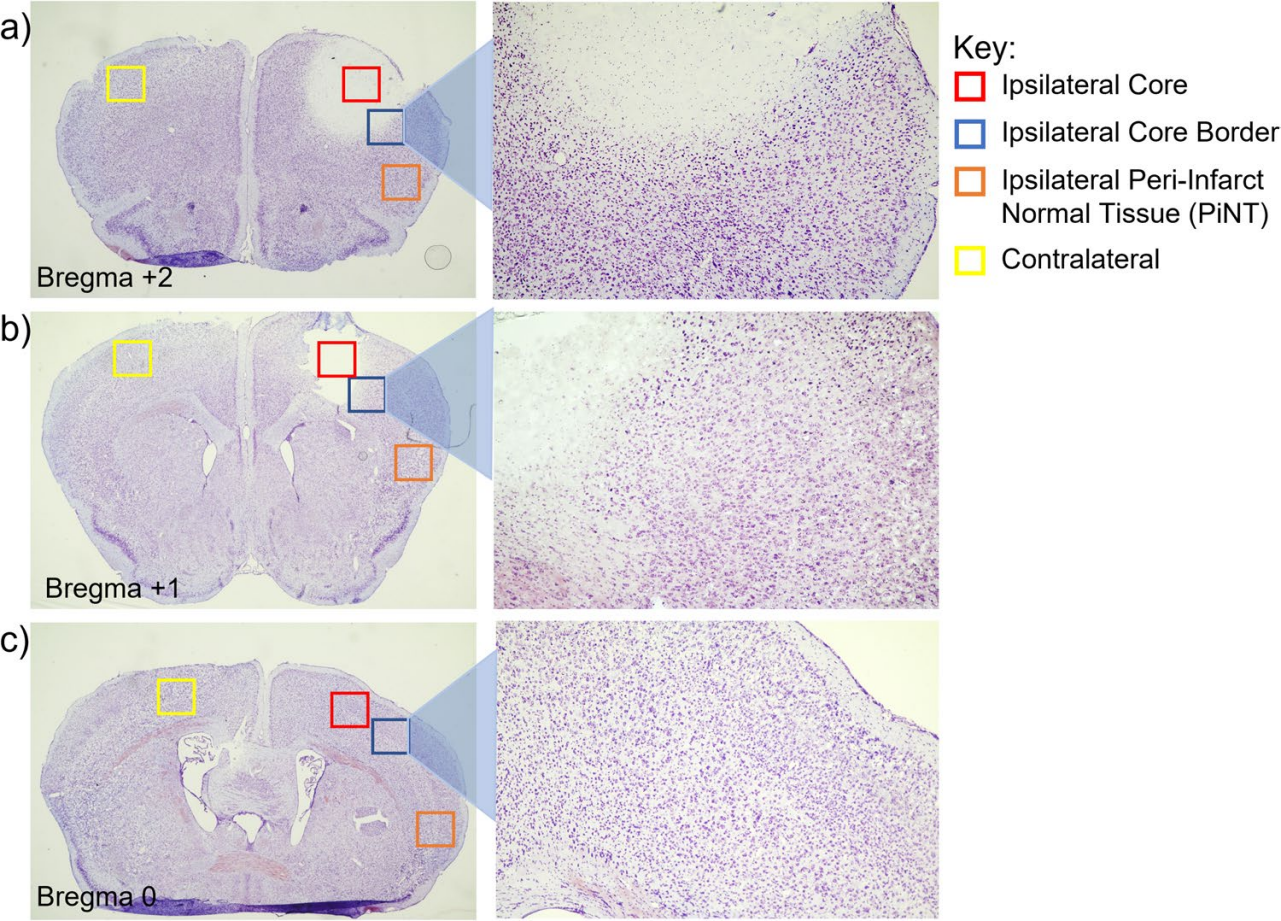
Representative stereotaxic locations and regions of interest at 3-days post-ischemia. Coronal sections of mice with induced photothrombotic MCAO were stained with cresyl violet at 3 days after MCAO and examined qualitatively at bregma + 2 (**a**) bregma + 1 (**b**) and bregma 0 (**c**) with four ROIs: ipsilateral core (red), ipsilateral core border (blue), ipsilateral peri-infarct normal tissue (PiNT; orange), and contralateral (yellow) with higher magnification of the core border region. Infarct at 3-days post-ischemia demonstrated larger core damage at bregma + 2 with some sustained damage at bregma + 1, but no indicated damage at bregma 0. Core border region demonstrates a small increase in cell numbers at both bregma + 2 and + 1

A Leica MZ FL III stereo microscope with a tethered dSLR camera was used to capture all digital images of cresyl violet stained sections between × 1.5 to × 2 magnification to capture a full tissue image and × 5 magnification to capture a clearer image of the core border region. Representative cresyl violet images were captured to demonstrate photothrombotic damage at each investigated stereotaxic location (bregma + 2, + 1, and 0) with highlighted focus on the core border region (Fig. 1).

A Leica DM5500 B Automated Upright fluorescence microscope was used to capture all digital images of FJB stained sections at × 5 magnification to capture the entire image area of FJB^+^ cells in the FITC channel at the same exposure time. Immunohistology images of FJB^+^ cells were captured at bregma + 2 and + 1. Images were taken at the ipsilateral core and corresponding contralateral cortical region of three separate tissue sections for each mouse (technical replicates = 3; 4 biological replicates). FJB^+^ cells were counted semi-automatically with ImageJ software (Media Cybernetics, Inc., Bethesda, MD) after threshold at size 10-infinity (pixel units) and circularity 0.4–1.00. FJB^+^ cell counts were averaged for each stereotaxic location.

A Nikon TS2-S-SM inverted fluorescence microscope equipped with a CCD camera was used to capture all digital images of Iba-1 and GFAP sections at × 10 magnification in the TRITC channel. Iba-1 and GFAP images were captured at the same exposure times, respectively for each marker. Immunohistology images of Iba-1, and GFAP were captured at approximately bregma + 2, + 1, and 0 at four different regions of interest: the core, core border, PiNT, and contralateral cortex in three separate corresponding tissue sections for each mouse (technical replicates = 3, 4 biological replicates). Mean gray values were calculated with ImageJ2 1.53d (Fiji) software. All picture properties were obtained and ensured for consistency. Pictures were converted to 32-bit gray before analysis, and pictures were not altered in any other way. Area fraction was utilized as a control where all picture values must have 100% area fraction. Mean gray value region of interest (ROI) technical replicates were normalized against the averaged mean gray value contralateral ROI. Iba-1 maxima count was analyzed with prominence = 15, in areas of high tissue damage (based on cresyl violet staining) where background signal may be higher, prominence was increased to 18. Maxima count ROI technical replicates were normalized against the averaged mean gray value contralateral ROI. Image collection and data analysis were performed by an individual who was blinded to the experimental conditions.

### Formalin-Fixed Paraffin-Embedded Tissue Preparation

Brains in preparation for Nanostring GeoMx DSP were continually dehydrated in preparation for paraffin embedding: 24 h of 4% PFA was followed by 24 h each in 40% ethanol, 70% ethanol, and a second change of 70% ethanol (MCAO, *n* = 3). Brains were then placed whole into cassettes and processed in a Core Facility Tissue-Tek Processor in a 12-h cycle before embedding coronally in paraffin. The 12-h cycle consisted of an initial 30-min ethanol wash, 5 × 1-h ethanol washes, 3 × 45-min incubation in CitriSolv, and 3 × 1-h incubation in paraffin wax before embedding (all reagents for the Tissue-Tek Processor were provided by the core facility).

Formalin-fixed, paraffin-embedded (FFPE) samples were sectioned to approximately bregma + 2 and 5-μm thickness sections were mounted onto slides and allowed to dry at room temperature overnight. FJB labeling was performed as described above to ensure infarct presence and record infarct size and location. Samples confirmed with FJB^+^ cells indicating successful MCAO were sent to Nanostring (Seattle, WA) for tissue preparation and GeoMx DSP profiling (*n* = 3 biological replicates). The GeoMx DSP Protein Assay protocol is described in detail in the GeoMx DSP Manual Slide Preparation User Manual (MAN-10150–01, page 12–26).

### Nanostring Digital Spatial Profiling Analysis

To visualize whole tissue, mounted slides were stained with oligo-conjugated antibodies for MAP2, Iba-1, and GFAP, and with Syto13 (nuclei) in the GeoMx DSP instrument. Circular geometric patterns (200 μm diameter) were used to identify six ROIs on the scanned tissues of each sample: core border, peri-infarct, and PiNT regions on ipsilateral and contralateral hemispheres. The GeoMx Neural Cell Profiling protein panel (73 proteins) was utilized for this analysis. UV-cleavable oligo-conjugated antibodies according to the panel were dispensed onto each ROI, UV-cleaved off, aspirated into a plate, hybridized, and counted by the GeoMx DSP instrument. All resulting spatial, quantified analysis was performed in the Nanostring GeoMx Data Analysis Suite software (v2.2). Background correction was determined by protein target correlation plot and high correlation was seen between Rb IgG, Rb IgGa, and Rb IgGb. All three IgG proteins were used for panel background correction via signal-to-background ratio. For detailed information on protein analyte validation, see the NanoString Whitepaper (MK2598): (https://nanostring.com/wp content/uploads/WP_GeoMx_Antibody_Validation_White_Paper.pdf).

### Pathway Analysis

For pathway analysis, we used the open-source web-based software Enrichr [30, 31] to analyze many databases at once and get a broad overview of important and over-represented pathways within our differentially expressed proteins. Z-score is calculated based upon the difference between the average rank for randomly chosen gene sets and the rank of the gene set of interest and is used to determine the degree of enrichment. Visualization of consistently highly represented pathways was done using pathway diagrams from the Kyoto Encyclopedia of Genes and Genomes (KEGG) from the most recent update of the database in 2019 [32]. Pathway diagrams were colorized using the Color tool in version 5 of KEGG Mapper [33].

### Statistical Analysis

Samples sizes were determined based on power calculations and findings from previous stroke studies performed at our laboratory [26]. Normalized mean gray values of ipsilateral ROIs from immunohistochemistry micrographs were compared against the contralateral ROIs in correlating bregma locations. Significance of the results was tested with a nested *t* test based on their normality of distribution (Shapiro–Wilk test) using Graphpad Prism 9 (GraphPad Software, San Diego, CA). *P* value < 0.05 was considered as significant. A Q-Q plot showed that the data were normally distributed (not shown) Gene expression analysis was performed using the GeoMx Data Analysis Suite with built-in statistical analysis (NanoString Technologies, Seattle, WA) according to manufacturer’s instructions. GeoMx DSP spatial ROIs were compared using a linear mixed model (LMM) with Bonferroni-Hochberg (BH) correction and random effect for Scan ID and ROI ID. Fold changes were identified by comparing the ROI/contralateral ROI with a significance of *p* value < 0.05.

## Results

### FluoroJadeB Identifies Ischemic Infarct 3 Days Post-MCAO

**Fig. 2:**
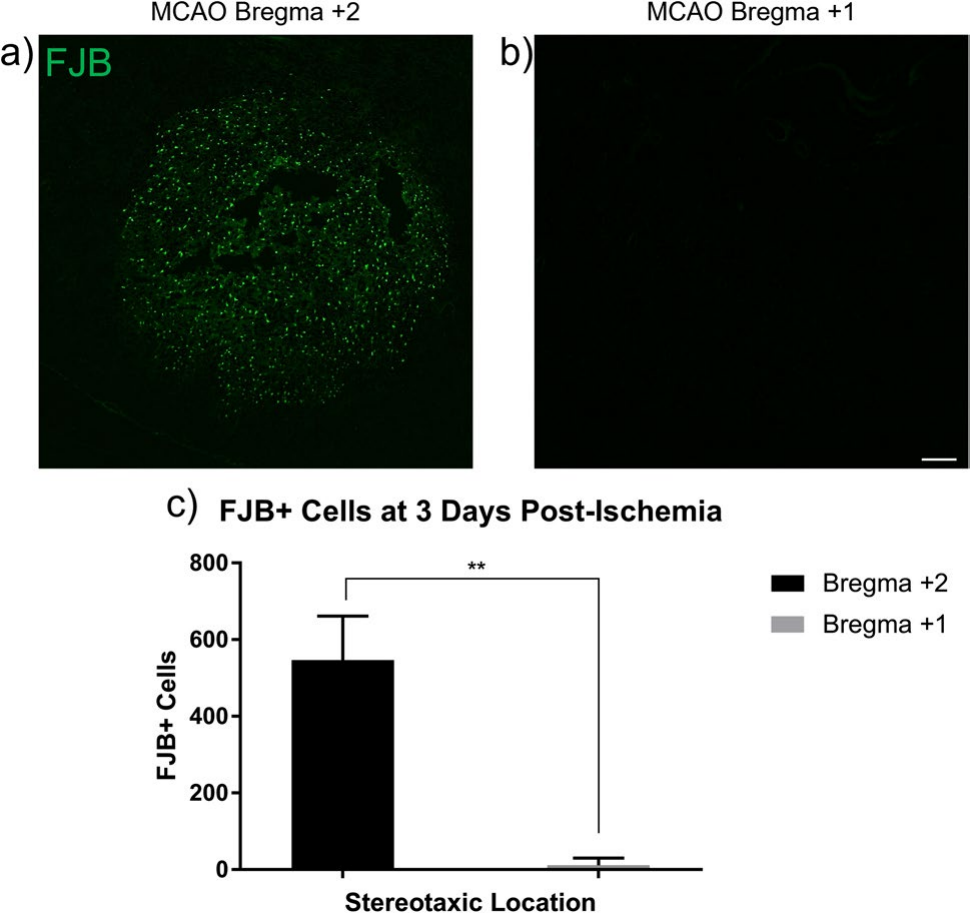
Neuronal damage 3 days post-ischemia. Brains from mice with induced MCAO were examined 3 days after ischemia. FJB^+^ cells were counted at bregma + 2 and + 1 at 5 × magnification for whole infarct comparison. Extensive FJB^+^ staining was found at bregma + 2 (**a**) but was not detected at bregma + 1 (*t*(6) = 9.253, *p* < 0.001) (**b**). A mean value of FJB^+^ cells from three brain sections from each location was obtained for each individual mouse brain (**c**; MCAO *n* = 4). Data are expressed as mean ± SD. Scale bar = 100 µm

We examined the consistency and ability to define the photothrombotic infarct at 3 days post-injury with the neurodegeneration stain, FJB. MCAO was induced in mice and brain tissue was collected and examined spatially at 3 days after ischemia at stereotaxic locations bregma + 2 and + 1. Detectable FJB^+^ cells were found consistently throughout the infarct core at bregma + 2, but not at bregma + 1 (Fig. 2). MCAO brain tissue exhibited a mean of 547.3 ± 114 FJB^+^ cells at bregma + 2 with only a mean of 11.6 ± 19 at bregma + 1. This demonstrated that neurodegenerative damage occurs and is detectable by FJB staining three days after MCAO with primary neuronal damage located at bregma + 2.

### GFAP Increases in Core Border 3 Days Post-Ischemia

**Fig. 3:**
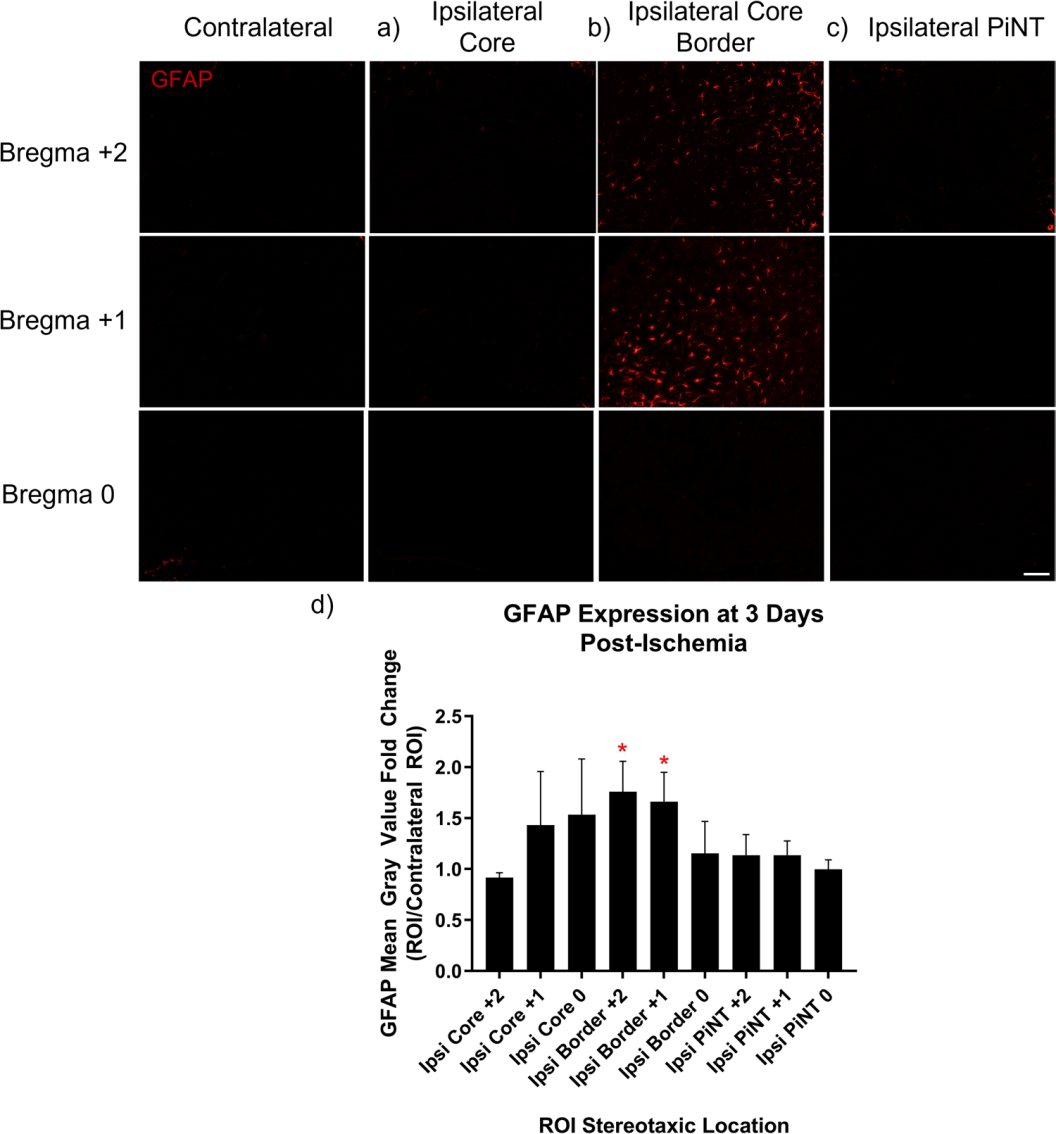
GFAP expression increases in core border spatially 3 days post-ischemia. Brains from mice with induced photothrombotic MCAO were examined spatially 3 days after MCAO for GFAP expression. Normalized mean gray value change of GFAP expression compared to the contralateral hemisphere in the ipsilateral core (**a**), ipsilateral core border (**b**), and ipsilateral PiNT (**c**). Mean gray value change increased significantly in the core at bregma + 2 (*t*(16) = 2.796, *p* = 0.013), core border at bregma + 2 (*t*(6) = 5.082, *p* = 0.002) and bregma + 1 (*t*(6) = 4.557, *p* = 0.004) but was non-significant in bregma 0 (**d**). Mean gray value fold change of GFAP was non-significant in PiNT in any stereotaxic location. Data are expressed as mean ± SD; *p* < 0.05, MCAO (*n* = 4). Scale bar = 100 µm

To characterize the early inflammatory processes that occur after MCAO, astrocytic immunoreactivity was assessed in a spatial manner with the immunohistology marker, GFAP. MCAO and sham controls (SHAM) were sacrificed at 3 days after ischemia and brain tissues were examined at bregma + 2, + 1, and 0 within the ipsilateral core, core border, PiNT, and contralateral cortex for GFAP expression. High GFAP expression is not typically observed in the naïve or SHAM mouse cortex but is upregulated within proliferating and activated astrocytes following neuronal injury [16, 34, 35]. Therefore, GFAP expression was analyzed as normalized mean gray value change against the contralateral ROI to detect regional changes in GFAP expression. GFAP regional expression changes in SHAM animals demonstrated no significant differences from contralateral ROI (data not shown). GFAP regional normalized mean gray value expression in MCAO animals was then examined throughout the ipsilateral hemisphere (Fig. 3). No significant changes in GFAP expression were seen in the ipsilateral core. GFAP expression significantly increased within the ipsilateral core border at both bregma + 2 and + 1 with a mean gray value expression change of 1.76 ± 0.15 and 1.66 ± 0.15, respectively. No significant changes in GFAP expression were seen in the PiNT region at any stereotaxic location. Additionally, GFAP mean gray value expression demonstrated no change at bregma 0 in all three regions. GFAP expression suggests that there is an increase in astrocytic inflammatory activity along the core border in a spatial manner at 3 days post-ischemia.

### Microglia Increase Reactivity Within Ischemic Region

**Fig. 4:**
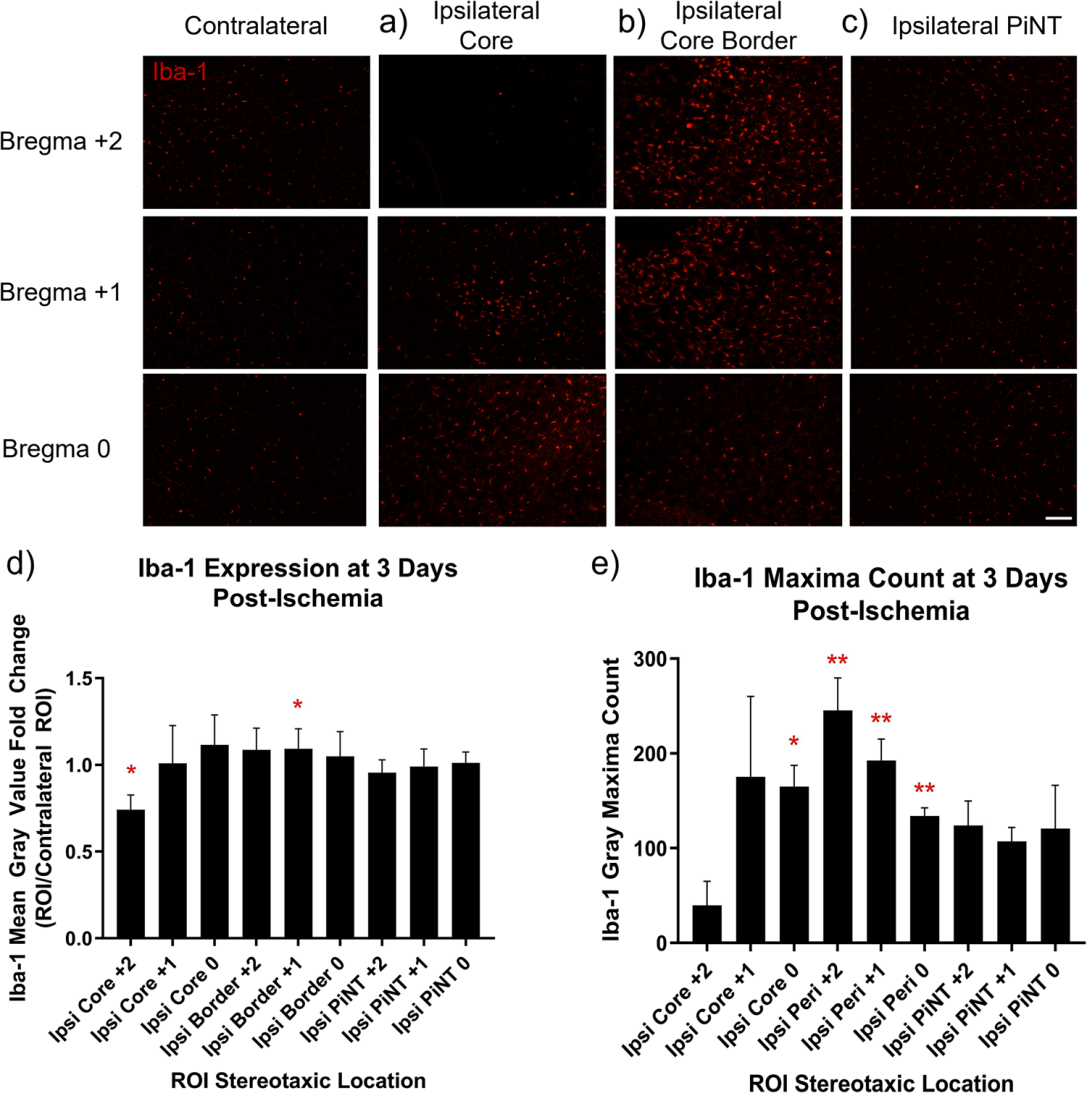
Iba-1 Expression Demonstrates Microglia Reactivity Spatially at 3 Days Post-Ischemia. Brains from mice with induced MCAO were examined spatially 3 days after ischemia for Iba-1 expression. Normalized mean gray value change and normalized gray maxima count of Iba-1 expression compared to the contralateral hemisphere in the ipsilateral core (**a**), ipsilateral core border (**b**), and ipsilateral PiNT (**c**). Normalized mean gray value (**d**) significantly decreased in core region at bregma + 2 (*t*(6) = 6.008, *p* = 0.001). Normalized mean gray value change of Iba-1 was non-significant in PiNT or in other regions of interest at any other stereotaxic location. Normalized gray maxima count of Iba-1 decreased in ipsilateral core region at bregma + 2 (*t*(6) = 6.502, *p* < 0.001) and ipsilateral PiNT at bregma + 1 (*t*(21) = 2.164, *p* = 0.042) but increased in the core region at bregma 0 (*t*(6) = 3.104, *p* = 0.021) and ipsilateral core border at bregma + 2 (*t*(6) = 3.626, *p* = 0.011) and + 1 (*t*(6) = 3.527, *p* = 0.012) (**e**). Data are expressed as mean ± SD; *p* < 0.05, MCAO (*n* = 4). Scale bar = 100 µm

To further characterize the inflammatory processes that occur after MCAO, microglial activity was assessed in a spatial manner with the immunohistology marker, Iba-1. MCAO and sham controls (SHAM) were sacrificed at 3 days after ischemia and brain tissues were examined spatially at bregma + 2, + 1, and 0 within the ipsilateral core, core border, PiNT, and contralateral cortex for Iba-1 expression. In the naive and SHAM mouse cortex, Iba-1 expressing microglia exhibit a quiescent, ramified morphology (Fig. 4) [16]. After ischemia, resident microglia can increase Iba-1 reactivity, change morphology, as well as infiltrating monocytes will express Iba-1 [36]. Iba-1 expression was analyzed as normalized mean gray value change and normalized gray maxima count against the contralateral ROI to detect regional changes in Iba-1 expression. Iba-1 regional expression changes and gray maxima count in SHAM animals demonstrated no significant differences from contralateral ROI (data not shown). When Iba-1 expression was compared as mean gray value expression change, Iba-1 expression was only seen to significantly decrease within the core region at bregma + 2 with a mean gray value change of − 0.26 ± 0.04 (Fig. 4d). No significant changes in Iba-1 normalized mean gray value changes were seen in any other ipsilateral regions at any other stereotaxic location. When Iba-1 Gy maxima count was measured, a more specific pattern of Iba-1 expression could be seen. Iba-1 normalized gray maxima count significantly decreased within the core region at bregma + 2 with a change of − 0.72 ± 0.11 (Fig. 4e). Iba-1 Gy maxima count also exhibited a significant increase within the core region at bregma 0 with a change of 0.35 ± 0.11. Immunohistology images (Fig. 4a) demonstrate a distinct morphological change in Iba-1 expressing cells within the bregma + 2 core region. There was also a disappearance of Iba-1 expressing cells from the center of the core region, likely reflecting within the decrease of gray values, specifically maxima count. Additionally, round Iba-1 expressing cells appear along the core border region with beginning invasion into the core, suggesting activation of residential microglia and potentially early invasion of peripheral monocytes.

**Fig. 5:**
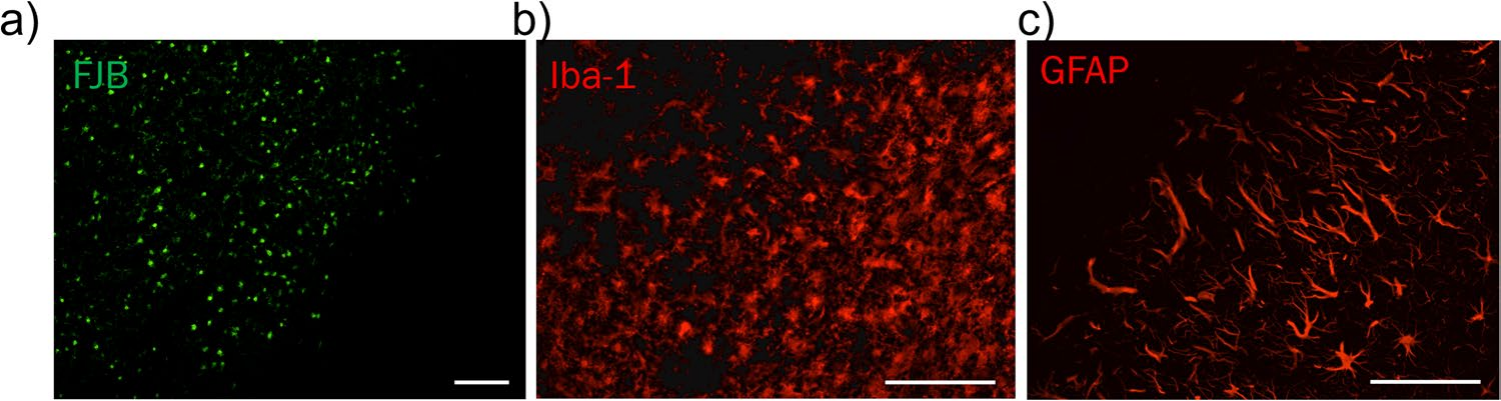
Histological markers define core border at 3 days post-ischemia. Immunohistology images of FJB (**a**; green), Iba-1 (**b**; red), and GFAP (**c**; red) compared at the core border at bregma + 2 can define a consistent, clear core border at 3 days post-ischemia. Scale bar = 100 µm

Iba-1 normalized gray maxima count demonstrates significantly increased Iba-1 counts at both core border bregma + 2 and + 1 with a change of 0.71 ± 0.20 and 0.47 ± 0.13, respectively (Fig. 4e). Iba-1 normalized gray maxima count was seen to be significantly decreased in the PiNT at bregma + 1 by − 0.20 ± 0.095, but no significant changes were seen in the PiNT at any other stereotaxic location. The increase in gray maxima count Iba-1^+^ round cells within the bregma core border location further suggests activation of residential microglia and early invasion of peripheral monocytes into the core in a spatial manner at three days post-ischemia. Inflammatory activation within the core border of GFAP- and Iba-1-expressing astrocytes and microglia demonstrates the importance of this region early after ischemia. Therefore, we have determined that consistent identification of the core border can be histologically defined with combination of these three glia cell stains: FJB, GFAP, and Iba-1 at 3 days post-ischemia (Fig. 5).

#### Nanostring Digital Spatial Profiling Defines Distinct MCAO Protein Profiles

**Fig. 6:**
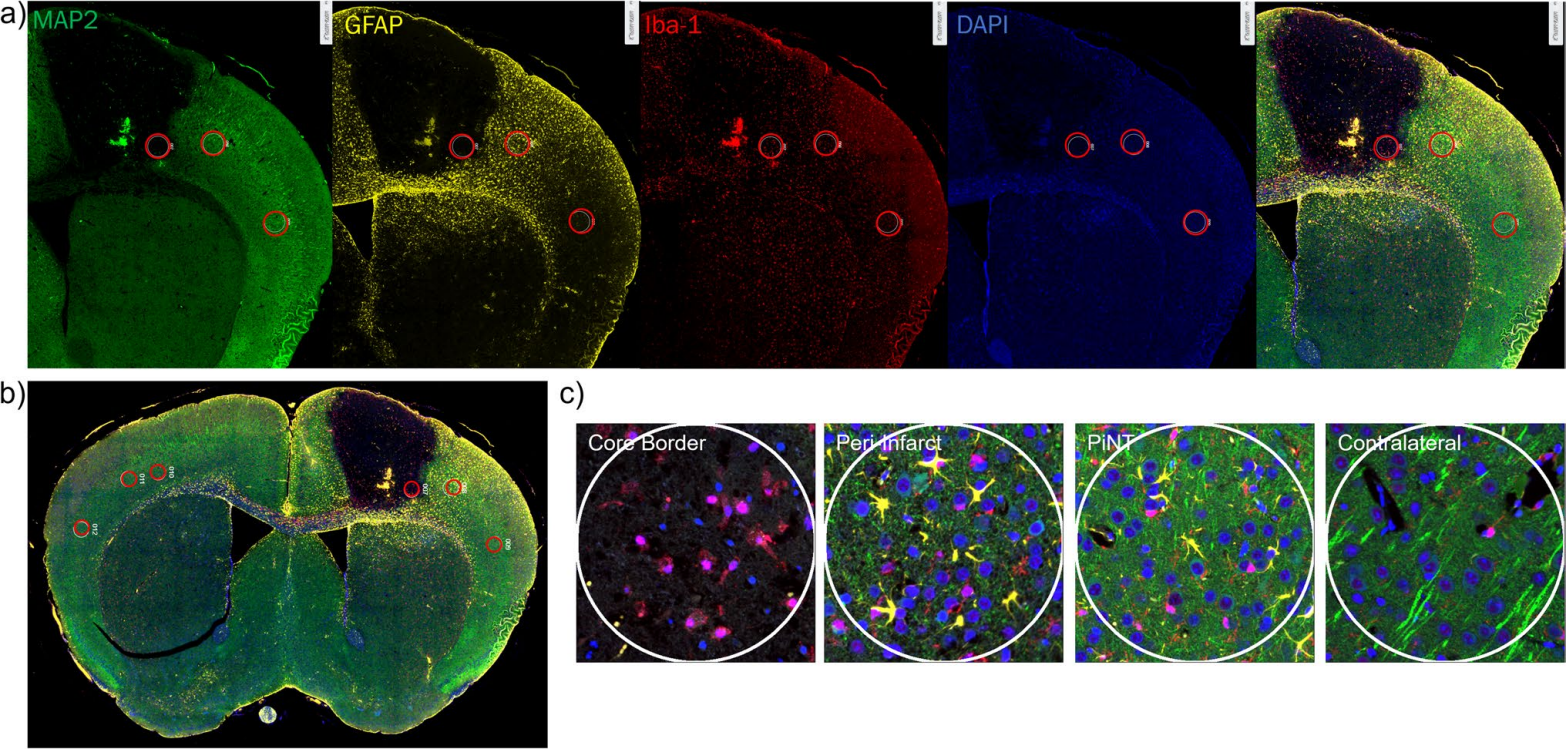
Representative immunohistochemical figures for DSP analysis and ROI selection. Coronal sections from mice with induced photothrombotic MCAO were immunohistologically stained for identifying markers MAP2 (green), GFAP (yellow), Iba-1 (red), and Syto13 for nuclei (blue) (**a**) and overlayed for ROI selection (**b**). Overlay of these markers clearly identified the extent of the infarct region and astroglia immunoreactivity within the core border. ROI’s included ipsilateral core border, ipsilateral peri-infarct, ipsilateral PiNT, and contralateral cortex, each region is outlined in a red circle (**c**). Clear differences in the identifying IHC markers can be seen within each ROI

In order to elucidate a detailed proteomic analysis and profile after ischemia within these specific ROI’s, we utilized Nanostring’s GeoMx DSP technology. MCAO mice were sacrificed at 3 days after ischemia and brain tissues were examined spatially in the ipsilateral core border, peri-infarct, and PiNT tissues compared to the contralateral side with the Nanostring GeoMx DSP Neural Cell Profiling protein panel (70 proteins; excluding IgG negative targets). Sections were immunostained with four chosen identifying markers for ROI selection (Fig. 6). Markers included MAP2 for neuronal damage, GFAP for astrocytic reactivity, Iba-1 for microglia, and Syto13 for nuclei. The full neural cell profiling protein panel was run on each ROI resulting in quantified protein read-outs for each ROI.

**Fig. 7:**
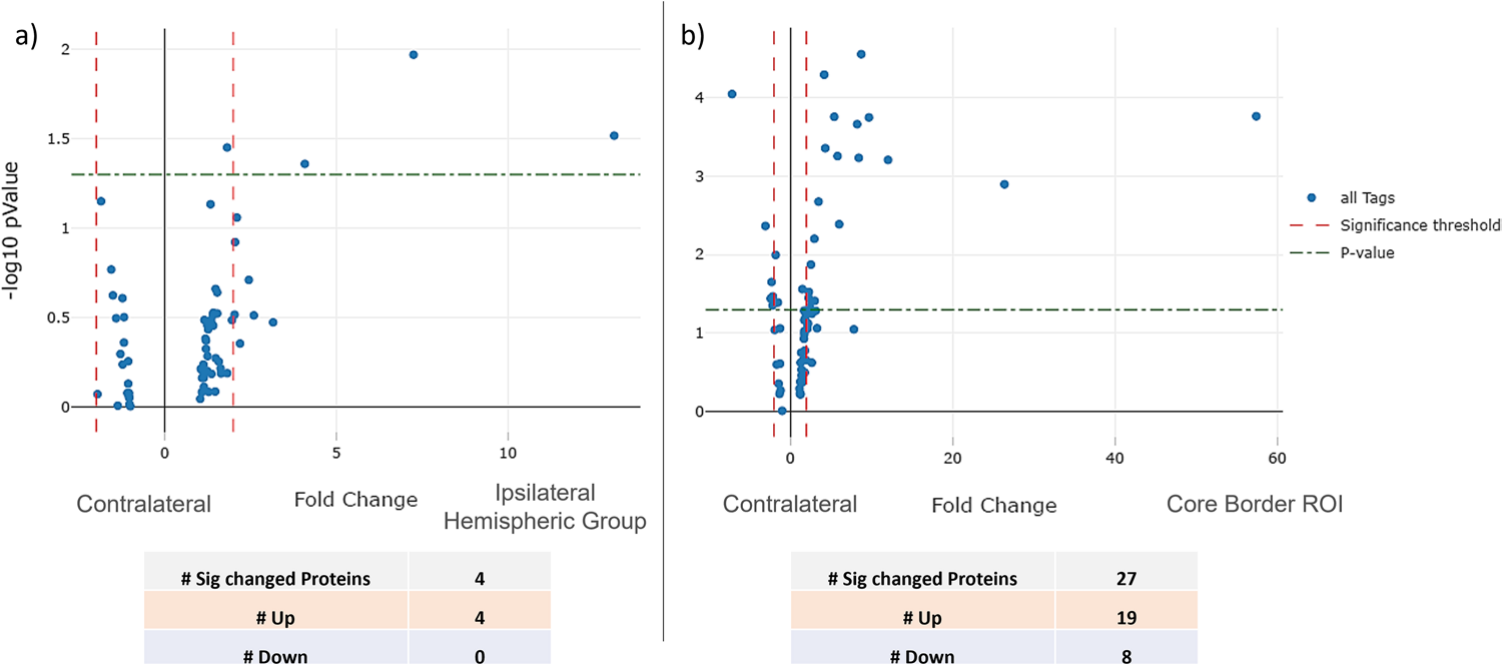
Ischemia presents region specific proteomic profiles. Volcano plots representing the significant protein fold changes of the ipsilateral hemispheric group (core border, peri-infarct, and PiNT ROIs) (**a**) and isolated core border ROI (**b**) compared against the contralateral ROI. Data is plotted on a -log10 *p* value scale; fold change on the *x*-axis; all tags (blue plotted dots) represent individual panel proteins; *p* value (0.05; green dotted × parallel line) (MCAO *n* = 3)

To determine the comparative significance of GeoMx DSP regional specific comparisons to whole tissue analysis, GeoMx DSP proteomic profiles were first compared as a combined ipsilateral hemispheric group to the contralateral hemisphere’s proteomic data. All ipsilateral ROIs (core border, peri-infarct, and PiNT ROIs) were combined as one ipsilateral hemispheric group and compared against the contralateral side for significantly changed proteins via an LMM. Then, the individual core ROI was compared against the contralateral side for significantly changed proteins via an LMM (Fig. 7). The whole ipsilateral hemispheric group resulted in only 4 significantly upregulated proteins. However, when the ipsilateral core border ROI was isolated and compared to the contralateral tissues, 27 proteins demonstrated significant changes, with 19 upregulated and 8 downregulated.

**Fig. 8:**
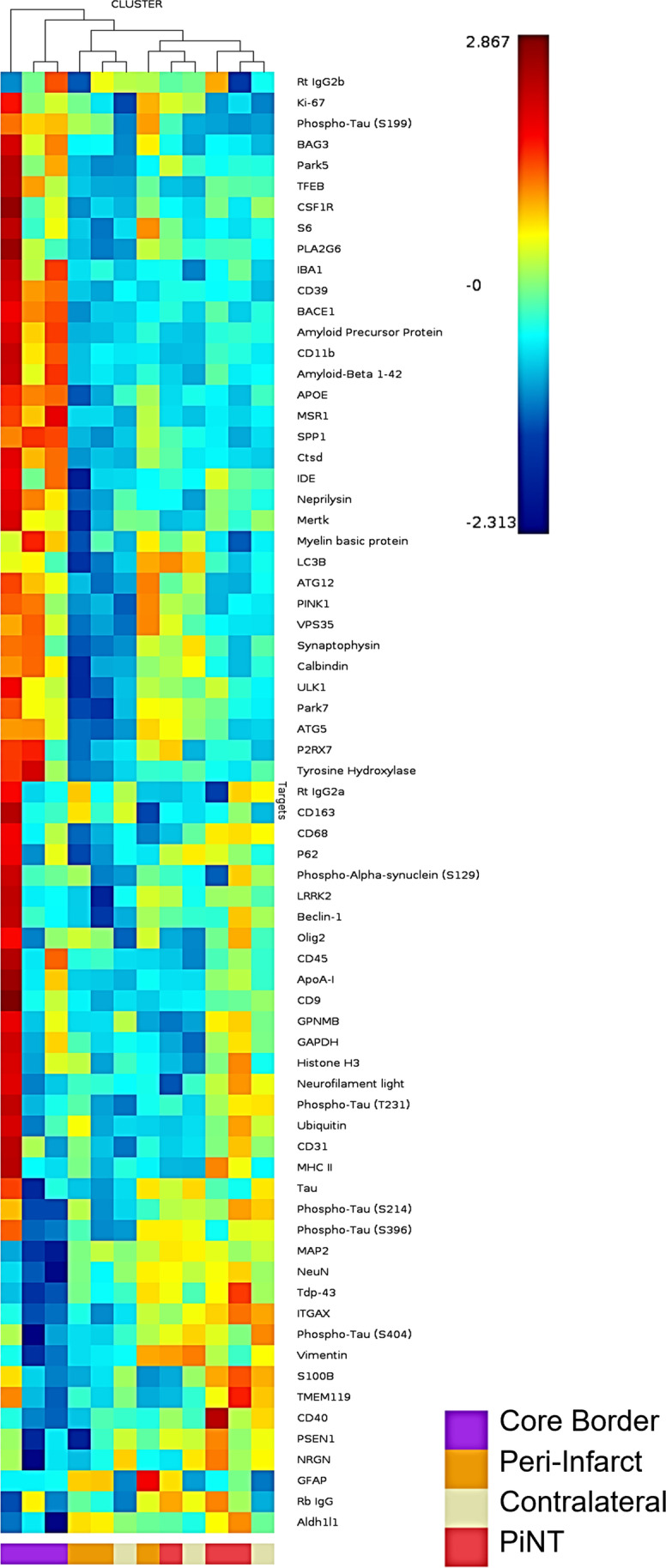
Hierarchical Cluster Heat Map of ROIs. Protein expression from each individual ROI representing the ipsilateral core border (purple), ipsilateral peri-infarct (orange), ipsilateral PiNT (red), and contralateral (white) is plotted on a heat map where red = higher representative expression and blue = lower representative expression. ROIs are clustered according to proteomic expression similarity where most similar are clustered together and tree relatives are mapped above

To determine general similarities and consistencies between the proteomic profiles of ROIs between biological samples, a hierarchical cluster was created (Fig. 8). Overall, all core border ROIs clustered together, demonstrating the most similar profiles across samples within the core border. Peri-infarct ROIs clustered closest to the core border ROIs, demonstrating similarity between samples within the peri-infarct region and closest proteomic profile next to the core border ROIs. The PiNT and contralateral ROIs clustered generally together outside of the core border and peri-infarct regions, demonstrating most similarity to each other between samples and furthest similarity to the core border and peri-infarct regions. Overall, this hierarchal cluster suggests that each ROI group demonstrates a unique, consistent proteomic profile after ischemia.

Each ROI was further characterized compared to the contralateral ROI. Significantly differentially regulated proteins according to fold change were documented for each ROI (Table 1). The ipsilateral core border ROI demonstrated 27 differentially regulated proteins with 19 upregulated and 8 downregulated. Of these 27 differentially regulated proteins, there was most notably a unique profile reflected in neuronal death, apoptotic, immunoreactive, and early degeneration proteins. Neuronal proteins, MAP2 and NeuN, resulted in downregulation of − 7.19 and − 3.07 respectively in the core border. An autophagy protein, BAG3, resulted in a 12.04-fold upregulation and a lysosomal autophagy protein, CTSD, was also upregulated by 8.44-fold. The astrocytic protein, Aldh1/1, was downregulated by − 2.30-fold and a glial cytoskeletal intermediate filament protein, vimentin, was downregulated by − 2.15-fold. Many proteins related to microglial immunoreactivity and immune peripheral invasion were significantly upregulated within the core border region. These included a 2.25-fold upregulation in CD45, a 4.31-fold upregulation in Iba-1, an 8.24-fold upregulation in CD11b, an 8.74-fold upregulation in CD39, and a 9.69-fold upregulation in MSR1. Notably, there was also a marked 26.39-fold upregulation in SPP1, a protein specifically associated with disease-related microglia.

**Table 1:**
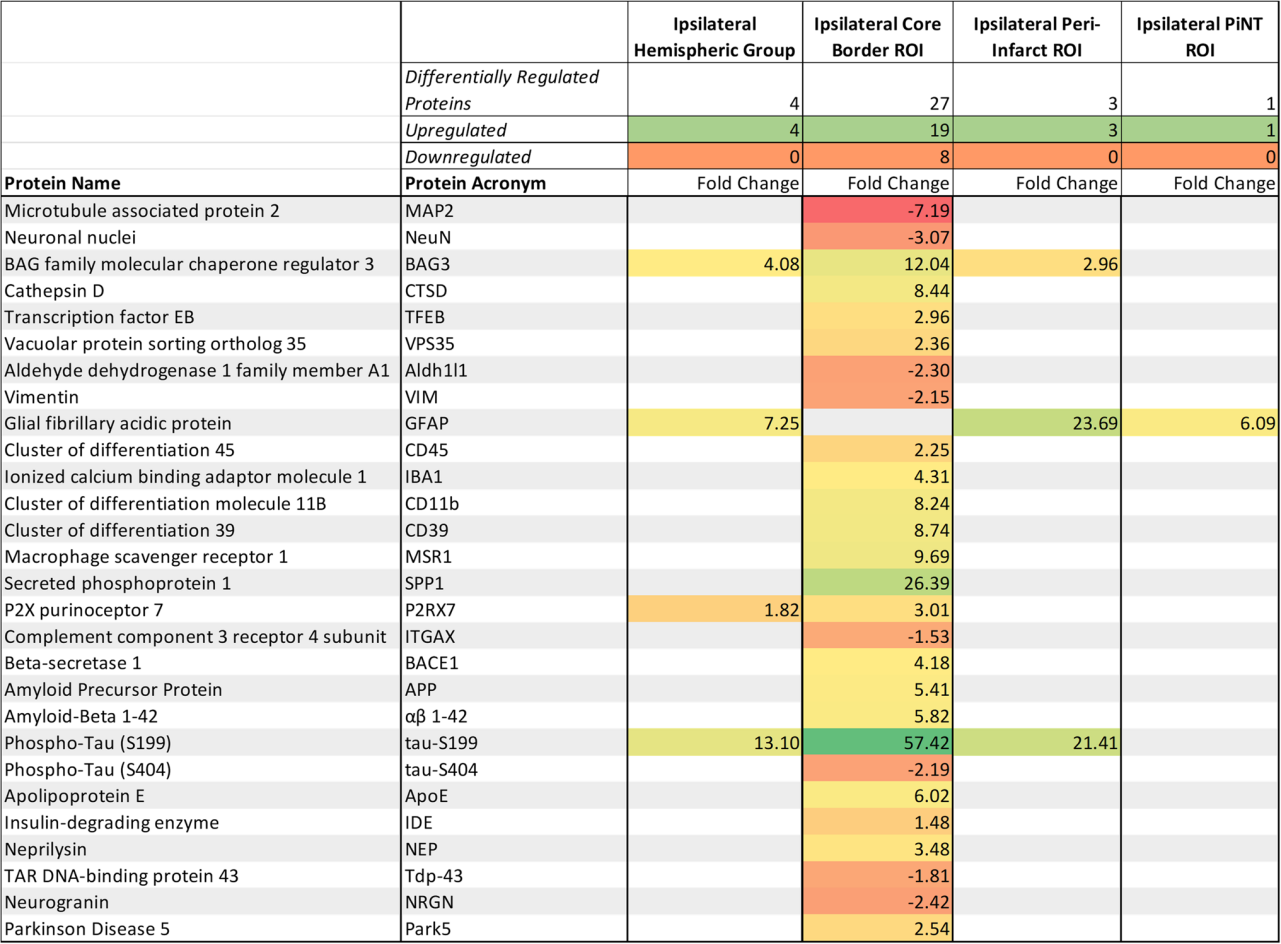
Regulatory Proteomic ROI Profiles. Proteins were compared against the contralateral side for significant fold changes via an LMM for each ROI including: ipsilateral hemispheric group, ipsilateral core border ROI, ipsilateral peri-infarct ROI, and ipsilateral PiNT ROI. Upregulated proteins expressed in yellow-green, downregulated proteins expressed in orange-red (*p* < 0.05, *n* = 3)

Within the core border region, BACE1 was upregulated by 4.18-fold, APP was upregulated by 5.41-fold, and amyloid β (αβ) 1–42 was upregulated by 5.82-fold. This suggests that 3 days after ischemia, the BACE1 cleaving protein was upregulated within the core and perhaps leads to increased cleaved products of APP and the αβ 1–42 form. Notably, there was a significant upregulation of the tau-S199 amyloid by 57.42-fold, which can lead to neuronal destabilization and death. There was also a significant upregulation of ApoE by 6.02-fold, which could later lead to be neuroprotective or neurodegenerative depending on whether it is expressing the neuroprotective isoform 3 or neurodegenerative isoform 4 [37]. The ipsilateral peri-infarct ROI demonstrated significant upregulation in 3 proteins: GFAP was upregulated by 23.69-fold, tau-S199 was upregulated by 21.41-fold, and BAG3 was upregulated by 2.96-fold. The ipsilateral PiNT ROI only demonstrated significant upregulation in one protein, GFAP, by 6.09-fold.

### Pathway Analysis

**Fig. 9:**
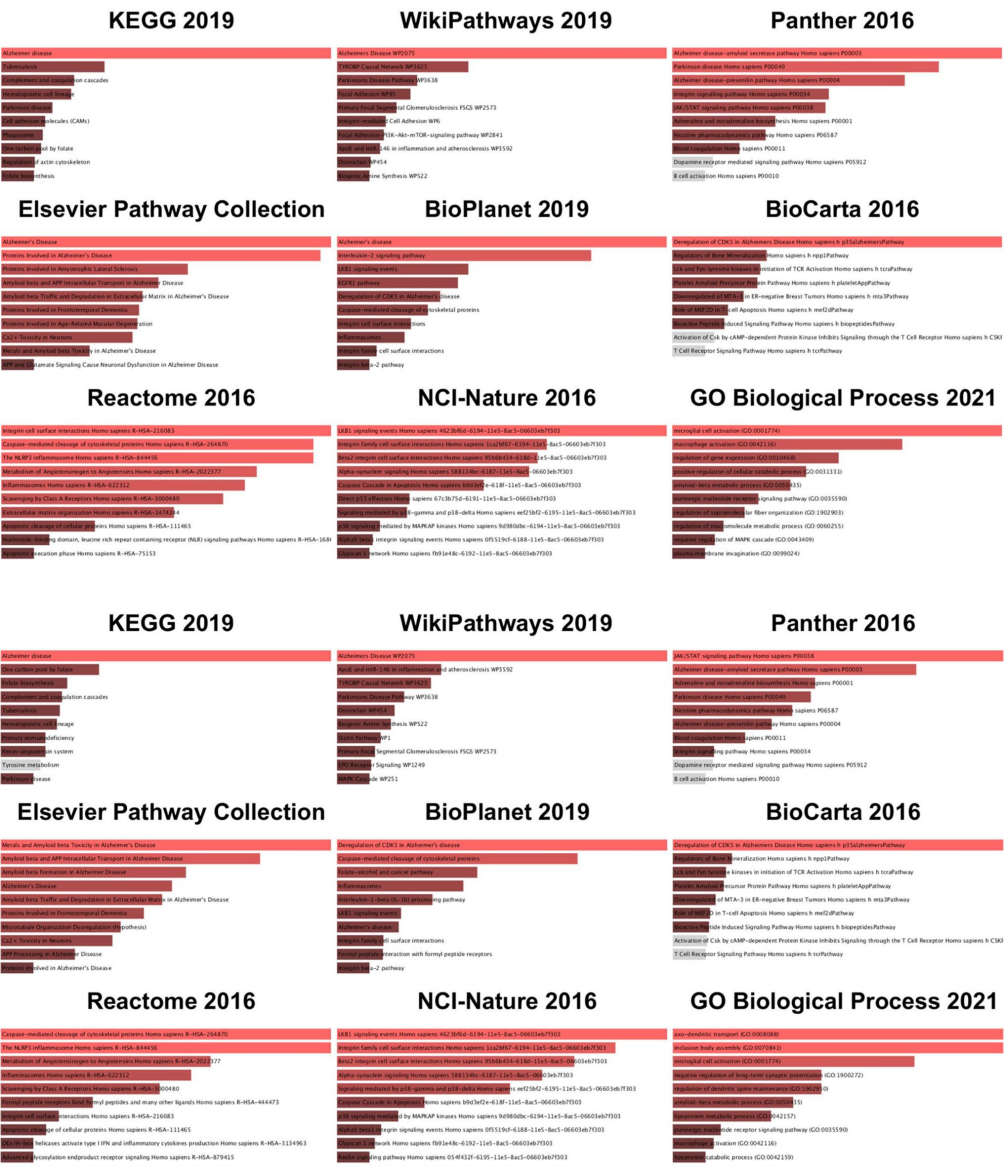
Most represented pathways and gene sets for differentially regulated proteins within the ipsilateral core border ROI. To determine the pathways with the most altered activity within the core border, overrepresentation analysis (ORA) was performed in Enrichr using the list of 27 differentially regulated proteins generated from LMM analysis. Top ten results of pathway ORA from nine different popular databases are shown. Upper panel shows results ranked by combined score, which integrates significance of result and magnitude of change. Lower panel ranks paths by p-value. Nine databases were used employing both ranking methods. For all bar graphs, length of the bar indicates relative magnitude of change. Red = significant (defined as FDR < 0.05); gray = not significant (FDR > 0.05)

**Fig. 10:**
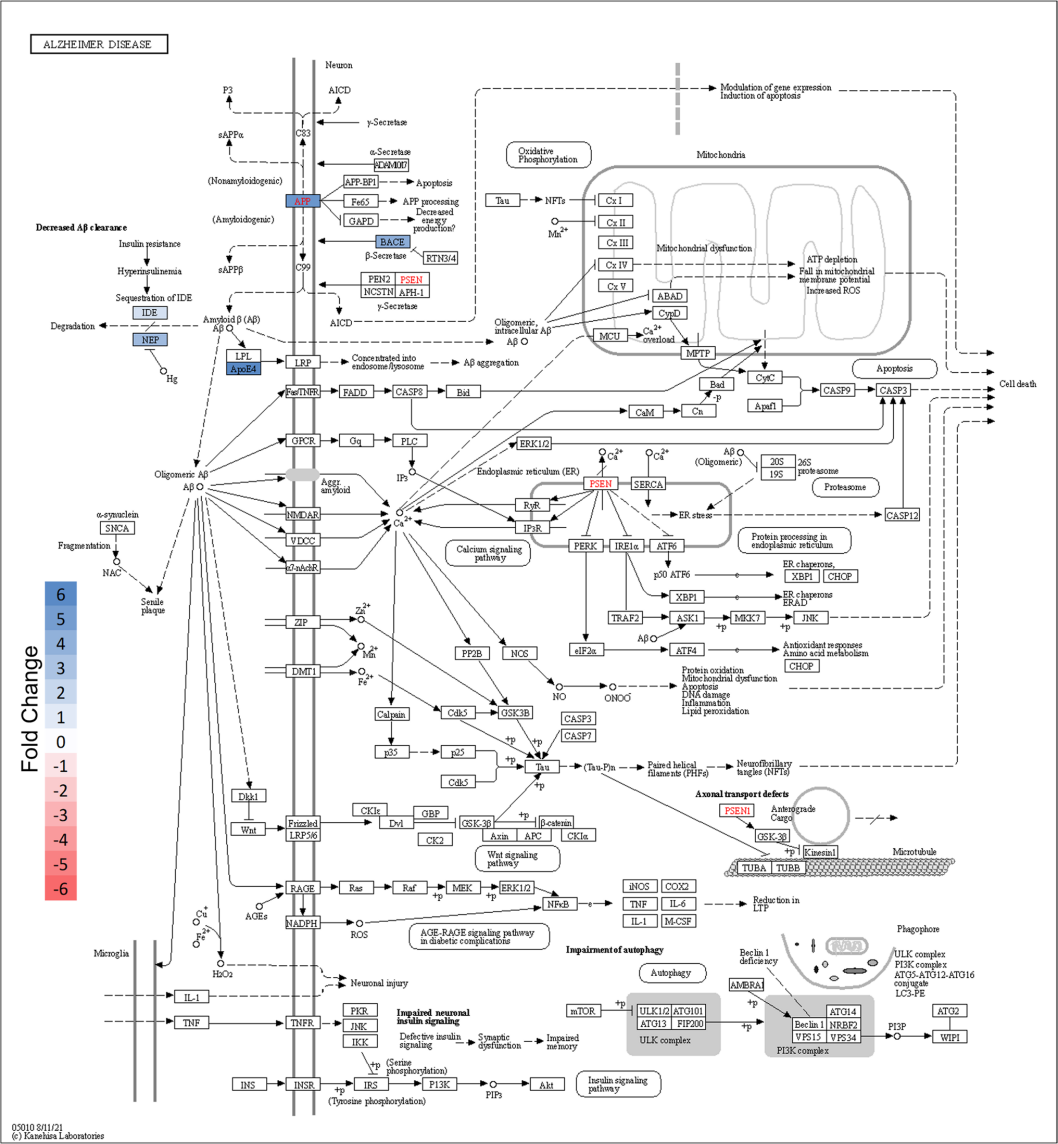

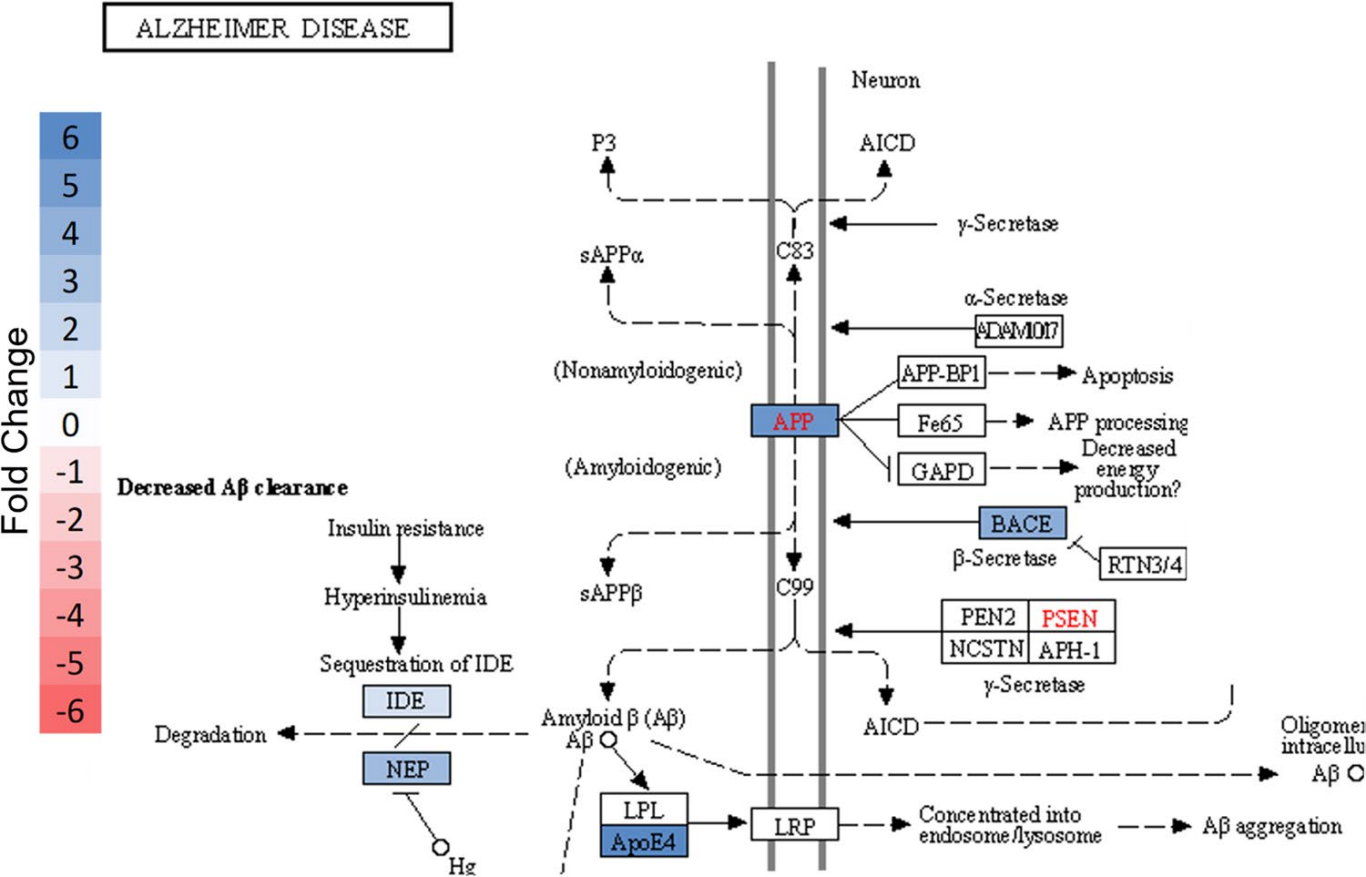
Alzheimer pathway regulation within core border ROI. Pathway diagram of Alzheimer disease obtained from KEGG database with color added to reflect significant protein expression level changes specific to the core border ROI (**a**). Protein colored based on significant (Blue = significant increase; FDR < 0.05) fold change compared to contralateral ROI. The most significant changes center around amyloid processing. An inset of the pathway region where proteins are altered after ischemia is shown (**b**)

To determine the pathways with the most altered activity within the core border, overrepresentation analysis (ORA) was performed in Enrichr using the list of 27 differentially regulated proteins generated from LMM analysis. The top ten results of pathway ORA from nine different popular databases are shown (Fig. 9). The upper panel shows results ranked by combined score, which integrates the significance of the result and the magnitude of change. The lower panel ranks pathways by *p* value. Over the nine databases and using both ranking methods, the most prominently overrepresented pathways were related to neurodegenerative diseases and the innate immune response. Alzheimer and related amyloid processing pathways were the most strongly enriched pathways. Other neurodegenerative pathways that were significant but lesser in magnitude of pathway activation included Parkinson’s disease and dementia. Innate immune activation is indicated by the strong enrichment of inflammasome pathways, integrin signaling pathways, caspase activation and related apoptotic pathways, and immune-cell specific signaling pathways including those for microglia and T cells. Examination of the Alzheimer pathway diagram indicates that the majority of activity change results from upregulation of proteins associated with amyloid processing (Fig. 10). Interestingly, we see an increase in both APP and BACE, which could lead to an increase in amyloid β fibrils, but also an increase in neprilysin (NEP) and insulin-degrading enzyme (IDE), both of which are enzymes responsible for amyloid degradation. APOE was the most dramatically upregulated Alzheimer pathway protein within the core border. APOE is a key mediator of Alzheimer progression as it triggers induction of amyloid β accumulation.

## Discussion

Our data presented emphasizes the importance of accounting for spatial differences when analyzing post-ischemic proteomic data. Our proteomic analysis presents unique profiles spatially and regionally in relation to the infarct core. Previous technologies using whole ipsilateral tissues can provide some insight into mechanisms occurring post-ischemia but can greatly obscure specific mechanisms that occur within each region. Our data demonstrated that each region analyzed with GeoMx DSP demonstrated a unique proteomic profile with distinctly regulated proteins. Within the ischemic core border, a unique profile relating to cellular death, immunoreactivity, and early degeneration was identified. When looking further into the relationship between these differentially regulated proteins, a potential pattern emerged with future interest for therapeutic targeting. As expected, the neuronal proteins, MAP2 and NeuN, were downregulated, indicative of neuronal death within the core border. This is also reflected in the upregulation of the autophagic protein, BAG3, and the lysosomal autophagy protein, CTSD. CTSD has been shown to increase sharply after ischemia but will decrease as lysosomes rupture [38]. BAG3 was also upregulated within the peri-infarct region, suggesting that autophagic block maybe ongoing within this region.

Ischemia also resulted in an upregulation of the general immune cell surface marker, CD45, as well as Iba-1. This suggests an increase in residential microglia and/or peripheral monocyte infiltration and activity. However, as residential microglia are recognized to decrease acutely within the core via degeneration, which is reflected within the immunohistochemistry mean gray value and gray maxima count changes, we expect this Iba-1 increase to reflect peripheral monocyte invasion [16, 36, 39]. This is also suggested by the eightfold upregulation of CD11b and CD39 and a threefold upregulation of the CD39 receptor, P2RX7, which are all surface markers on leukocytes. Leukocytes are noted to begin infiltrating into the ischemic region as early as 12 h after ischemia and persist, with peak levels at 7 days post-ischemia [16, 17, 36]. Microglia reactivity post-ischemia is notoriously recognized to have both beneficial and deleterious effects. Activated microglia acutely can phagocytose cells that have necrotized within the lesion core, preventing the further release of neurotoxic products [39–41]. However, activated microglia also release many pro-inflammatory cytokines and reactive oxygen species which can contribute to infarct development [39–41]. Therefore, truly understanding the interactions of these cytokines and proteins is key to identifying future targets for therapeutic intervention. For example, CD39 and MSR1 both demonstrated an upregulation of approximately ninefold, and both proteins have been suggested to play a neuroprotective role within the inflammatory process. Transgenic mouse lines overexpressing CD39 resulted in reduced leukocyte infiltration, smaller infarct volumes, and decreased neurological deficit after ischemia, suggesting a neuroprotective role of CD39 during ischemic insult [42]. CD39 expression on endothelial cells and leukocytes is suggested to reduce inflammatory cell trafficking and platelet reactivity via cell–cell interaction after ischemia [43]. Also, it has been shown that higher expression of the MSR1 protein increases damage-associated molecular patterns (DAMP) clearance after ischemia via MSR1-induced resolution of neuroinflammation, indicating MSR1 as a potential therapeutic target [44]. Additionally, there was a notable 57-fold upregulation of the protein SPP1. This protein is suggested to function as a pro-angiogenic trophic factor, but it is also associated with neurodegeneration as it is recorded to be upregulated in senescent microglia in an age-dependent manner [45, 46]. Controversially, this protein has also been associated with macrophagic communication to astrocytic migration toward the infarct area that has been suggested to be a neuroprotective repair mechanism of the ischemic neurovascular unit early after ischemia [47].

A unique astrocytic proteomic profile is reflective throughout each region. Within the core border, there is downregulation of the astrocytic proteins, Aldh1/1 and vimentin by about twofold. Vimentin-expressing astrocytes have been shown to increase acutely after ischemia but given downregulation at this 3-day timepoint in combination with Aldh1/1 downregulation, may suggest astrocytic degeneration within the core [48]. However, large upregulation of GFAP within the peri-infarct region by almost 24-fold and confirmed with mean gray value change increase at the core border both at bregma + 2 and + 1 suggests strong reactivity of astrocytes within the border and peri-infarct region 3 days after ischemia. GFAP has been shown by multiple studies to be increased specifically within the peri-infarct region beginning as early as 4 h after ischemia up to 28 days [16, 17, 48]. Reactivity of astrocytes and microglia can lead to secondary tissue damage, progressive cavitation, and scar formation along the peri-infarct border [49]. Ultimately, the relative differences between these microglial and astrocytic proteomic profiles within the core border and peri-infarct regions could provide potential targets for prevention of secondary damage and anti-inflammation.

Ischemia is well-recognized to be associated with neurodegeneration and more specifically the relationship between stroke history, tau proteins, and increased Alzheimer disease prevalence [50, 51]. Multiple tau-related proteins within the core border region resulted in a pattern that could ultimately indicate a future therapeutic target. BACE1, APP, and amyloid β 1–42 all demonstrated an approximate five-fold upregulation within the core border. BACE1 has been shown to be upregulated in response to released reactive oxygen species and hypoxia inducible factor 1α (HIF1α) following ischemia [50, 52]. Increased BACE1 activity results in increased cleavage of APP with increased deposits and aggregation of the neurotoxic form of amyloid β 1–42 [50, 52]. BACE1 expression was found to be regulated by a positive feedback loop between γ- and β-secretase cleavages on APP [53, 54]. Additionally, upregulation of APP within neurons, astrocytes, or microglia can lead to neuronal destabilization and vulnerability to stress, suggesting that upregulation of all three proteins within the core border could be indicative of a neurodegenerative profile [37]. The tau-S199 form was also found highly upregulated by 57-fold within the core border, and this tau form has been shown to play a role in the development of Alzheimer-like lesions after ischemia. After ischemia, tau proteins become rapidly dephosphorylated acutely and are then slowly rephosphorylated and accumulated in a serine site-specific manner [51]. Tau-S199 has been reported to be specifically induced after ischemia and to contribute to ischemic neuronal injury [51]. Dysregulation and displacement of tau proteins after ischemia is suggested to contribute to a neurodegenerative profile. Lastly, upregulation of the protein ApoE within the core border region could play a dual role depending on the isoform of ApoE that is being expressed. ApoEε3 is the most common isoform and can exhibit a neuroprotective effect by contributing to communication from microglia to neurons for repair, regeneration, and survival as well as suppression of microglia reactivity [37]. However, the ApoEε4 isoform may be neurodegenerative by contributing to amyloid β deposition [37].

## Conclusions and Future Directions

The ability to isolate the ipsilateral core border, ipsilateral peri-infarct, ipsilateral PiNT, and contralateral cortex regions and compare their proteomic profiles in such a detailed manner has allowed us to determine that each region demonstrates a unique differentially regulated proteomic profile. Future ischemia studies could greatly benefit from spatial analysis as we have demonstrated the substantial loss of data that can occur with whole tissue analysis. We examined proteomic profiles at 3 days post-ischemia to determine if stroke produced a neurogenerative profile after stroke that could be reversed by therapeutic intervention to stimulate post-injury repair. We are currently investigating protein profiles at different time points post-ischemia during the subacute phase (24 h to 5 days). Additional studies are ongoing to determine the spatiotemporal profile during the acute phase of stroke (less than 24 h) to explore mechanisms of early neurotoxicity and endogenous neuroprotection. However, the GeoMx DSP protein assay does have limitations such as limited plex and restriction to user selected regions of interest. To overcome this, we will determine spatiotemporal gene expression using NanoString’s GeoMx DSP Whole Transcriptome Atlas (WTA) RNA assay which will examine ~ 20 k transcripts occurring in our ROIs at different timepoints post-ischemia, with different MCAO models and with treatment paradigms. We will also perform these studies with transgenic mice generated to express unique channel fluorescence for resident microglia vs infiltrating and perivascular monocytes/macrophages which will allow us to utilize the GeoMx DSP segmentation function to isolate each cell type for spatiotemporal transcriptomic and proteomic changes post-ischemia. Additionally, NanoString is releasing the CosMx, an in situ single-cell platform that will be capable of analyzing tissue at a cellular and subcellular level with 1000 RNA plex, to further analyze cell-to-cell interaction and cellular processes. We will utilize our GeoMx DSP data to determine which timepoints and MCAO models demonstrate the most dynamic cell activity and analyze those with the CosMx technology. Overall, our data highlight the importance of identifying ischemic spatial mechanisms to understand the complex, dynamic interactions throughout ischemic progression, and repair as well to introduce potential targets for successful ischemic therapeutic interventions.

## Data Availability

Data sets generated and analyzed in this study will be deposited into the National Center for Biotechnology Information Gene Expression Omnibus (http://www.ncbi.nlm.nih.gov/geo) upon acceptance of the manuscript.

## References

[1] Benjamin EJ et al (2017) Heart Disease and Stroke Statistics-2017 Update: A report from the American Heart Association (vol 135, pg e146, 2017). Circulation 136(10):E196-E196 10.1161/CIR.0000000000000485 PMC5408160 28122885

[2] Fisher M (2009). Update of the stroke therapy academic industry roundtable preclinical recommendations. Stroke.

[3] Bosetti F (2017). Translational stroke research: vision and opportunities. Stroke.

[4] Detante O (2014). Biotherapies in stroke. Rev Neurol (Paris).

[5] Jayaraj RL (2019). Neuroinflammation: friend and foe for ischemic stroke. J Neuroinflammation.

[6] Jeong HK (2013). Brain inflammation and microglia: facts and misconceptions. Exp Neurobiol.

[7] Hossmann KA (2006). Pathophysiology and therapy of experimental stroke. Cell Mol Neurobiol.

[8] Brouns R, De Deyn PP (2009). The complexity of neurobiological processes in acute ischemic stroke. Clin Neurol Neurosurg.

[9] Lipton P (1999). Ischemic cell death in brain neurons. Physiol Rev.

[10] Price CJ (2006). Intrinsic activated microglia map to the peri-infarct zone in the subacute phase of ischemic stroke. Stroke.

[11] Cramer SC (2006). Activity in the peri-infarct rim in relation to recovery from stroke. Stroke.

[12] Hallenbeck JM (1996). Significance of the inflammatory response in brain ischemia. Acta Neurochir Suppl.

[13] Kim JY (2016). Inflammation after ischemic stroke: the role of leukocytes and glial cells. Exp Neurobiol.

[14] Pekny M, Nilsson M (2005). Astrocyte activation and reactive gliosis. Glia.

[15] Kawabori M, Yenari MA (2015). The role of the microglia in acute CNS injury. Metab Brain Dis.

[16] Nowicka D (2008). Spatiotemporal dynamics of astroglial and microglial responses after photothrombotic stroke in the rat brain. Acta Neurobiol Exp (Wars).

[17] Sanchez-Bezanilla S et al (2021) More than motor impairment: a spatiotemporal analysis of cognitive impairment and associated neuropathological changes following cortical photothrombotic stroke. J Cereb Blood Flow Metab 271678X211005877 10.1177/0271678X211005877 PMC8393292 33779358

[18] Surles-Zeigler MC et al (2018) Transcriptomic analysis of neuregulin-1 regulated genes following ischemic stroke by computational identification of promoter binding sites: A role for the ETS-1 transcription factor. Plos One 13(6) 10.1371/journal.pone.0197092 PMC5983438 29856744

[19] Xu Z (2005). Neuroprotection by neuregulin-1 following focal stroke is associated with the attenuation of ischemia-induced pro-inflammatory and stress gene expression. Neurobiol Dis.

[20] Ford G (2006). Expression analysis systematic explorer (EASE) analysis reveals differential gene expression in permanent and transient focal stroke rat models. Brain Res.

[21] Xu Z (2004). Neuregulin-1 is neuroprotective and attenuates inflammatory responses induced by ischemic stroke. Biochem Biophys Res Commun.

[22] Rodriguez-Mercado R (2012). Acute neuronal injury and blood genomic profiles in a nonhuman primate model for ischemic stroke. Comp Med.

[23] Simmons LJ et al (2016) Regulation of inflammatory responses by neuregulin-1 in brain ischemia and microglial cells in vitro involves the NF-kappa B pathway. J Neuroinflammation 13 10.1186/s12974-016-0703-7 PMC5011915 27596278

[24] Wang SL (2015). Spatio-temporal assessment of the neuroprotective effects of neuregulin-1 on ischemic stroke lesions using MRI. J Neurol Sci.

[25] Xu Z (2006). Extended therapeutic window and functional recovery after intraarterial administration of neuregulin-1 after focal ischemic stroke. J Cereb Blood Flow Metab.

[26] Noll JM (2019). Neuroprotection by exogenous and endogenous neuregulin-1 in mouse models of focal ischemic stroke. J Mol Neurosci.

[27] Gupta S (2020). Digital quantitative assessment of PD-L1 using digital spatial profiling. Lab Invest.

[28] Agrawal P (2015). The psychosocial factors related to obesity: a study among overweight, obese, and morbidly obese women in India. Women Health.

[29] Zhong J (2010). Hydrogel matrix to support stem cell survival after brain transplantation in stroke. Neurorehabil Neural Repair.

[30] Chen EY (2013). Enrichr: interactive and collaborative HTML5 gene list enrichment analysis tool. BMC Bioinforma.

[31] Kuleshov MV (2016). Enrichr: a comprehensive gene set enrichment analysis web server 2016 update. Nucleic Acids Res.

[32] Kanehisa M, Sato Y (2020). KEGG Mapper for inferring cellular functions from protein sequences. Protein Sci.

[33] Kanehisa M, Sato Y, Kawashima M (2021) KEGG mapping tools for uncovering hidden features in biological data. Protein Sci 10.1002/pro.4172 PMC8740838 34423492

[34] Davies CA (1998). An integrated analysis of the progression of cell responses induced by permanent focal middle cerebral artery occlusion in the rat. Exp Neurol.

[35] Savchenko VL (2000). Microglia and astrocytes in the adult rat brain: comparative immunocytochemical analysis demonstrates the efficacy of lipocortin 1 immunoreactivity. Neuroscience.

[36] Ito D (2001). Enhanced expression of Iba1, ionized calcium-binding adapter molecule 1, after transient focal cerebral ischemia in rat brain. Stroke.

[37] Koistinaho M, Koistinaho J (2005). Interactions between Alzheimer's disease and cerebral ischemia–focus on inflammation. Brain Res Brain Res Rev.

[38] Liu Y (2019). Neuronal-targeted TFEB rescues dysfunction of the autophagy-lysosomal pathway and alleviates ischemic injury in permanent cerebral ischemia. Autophagy.

[39] Matsumoto H (2007). Antibodies to CD11b, CD68, and lectin label neutrophils rather than microglia in traumatic and ischemic brain lesions. J Neurosci Res.

[40] Giulian D, Vaca K, Corpuz M (1993). Brain glia release factors with opposing actions upon neuronal survival. J Neurosci.

[41] Giulian D (1993). Reactive mononuclear phagocytes release neurotoxins after ischemic and traumatic injury to the central nervous system. J Neurosci Res.

[42] Baek AE (2017). Ischemic cerebroprotection conferred by myeloid lineage-restricted or global CD39 transgene expression. Circulation.

[43] Hyman MC (2009). Self-regulation of inflammatory cell trafficking in mice by the leukocyte surface apyrase CD39. J Clin Invest.

[44] Zou X (2021). Neuroprotective effect of phthalide derivative CD21 against ischemic brain injury: involvement of MSR1 mediated DAMP peroxiredoxin1 clearance and TLR4 signaling inhibition. J Neuroimmune Pharmacol.

[45] Li SL (2010). An age-related sprouting transcriptome provides molecular control of axonal sprouting after stroke. Nat Neurosci.

[46] Jiang L (2020). Transcriptomic and functional studies reveal undermined chemotactic and angiostimulatory properties of aged microglia during stroke recovery. J Cereb Blood Flow Metab.

[47] Choi IA et al. (2019) Sequential transcriptome changes in the penumbra after ischemic stroke. Int J Mol Sci. 20(24) 10.3390/ijms20246349 PMC6940916 31888302

[48] Kindy MS, Bhat AN, Bhat NR (1992). Transient ischemia stimulates glial fibrillary acid protein and vimentin gene expression in the gerbil neocortex, striatum and hippocampus. Brain Res Mol Brain Res.

[49] Fitch MT (1999). Cellular and molecular mechanisms of glial scarring and progressive cavitation: in vivo and in vitro analysis of inflammation-induced secondary injury after CNS trauma. J Neurosci.

[50] Sun X (2006). Hypoxia facilitates Alzheimer's disease pathogenesis by up-regulating BACE1 gene expression. Proc Natl Acad Sci U S A.

[51] Zheng GQ (2010). Tau as a potential novel therapeutic target in ischemic stroke. J Cell Biochem.

[52] Guglielmotto M (2009). The up-regulation of BACE1 mediated by hypoxia and ischemic injury: role of oxidative stress and HIF1alpha. J Neurochem.

[53] Wang R (2006). Transcriptional regulation of APH-1A and increased gamma-secretase cleavage of APP and Notch by HIF-1 and hypoxia. FASEB J.

[54] Li L (2009). Hypoxia increases Abeta generation by altering beta- and gamma-cleavage of APP. Neurobiol Aging.

